# ChatGPT‐Assisted Image Interpretation for Inflammatory Bowel Diseases: Ulcerative Colitis and Crohn's Disease

**DOI:** 10.1002/jgh3.70396

**Published:** 2026-04-02

**Authors:** Hiroki Uekado, Daisuke Watanabe, Yuichiro Aoyama, Hina Kawase, Sayaka Ikeda, Aya Shiraki, Jessica Pajimna, Misaki Agawa, Hirotaka Nakamura, Yuki Ito, Norihiro Okamoto, Haruka Miyazaki, Yuna Ku, Makoto Ooi, Namiko Hoshi, Yuzo Kodama

**Affiliations:** ^1^ Division of Gastroenterology, Department of Internal Medicine Kobe University Graduate School of Medicine Kobe Japan; ^2^ Institute of Digestive and Liver Diseases St. Luke's Medical Center Quezon City Philippines

## Abstract

**Objective:**

Differentiating ulcerative colitis (UC) from Crohn's disease (CD) is challenging, particularly for nonexperts. Although artificial‐intelligence‐based image analysis has advanced endoscopic diagnosis, large language models of inflammatory bowel disease (IBD) require clinical validation. We evaluated the ability of ChatGPT to distinguish UC from CD using colonoscopy (CS) images with and without clinical information.

**Methods:**

We retrospectively analyzed 386 and 161 patients with UC and CD, respectively, with active disease who underwent CS between April 2001 and May 2025. A representative endoscopic image showing severe activity at the initial flare was selected by a nonspecialist. Data were collected on lesion continuity and perianal disease. ChatGPT was asked to (1) classify UC or CD and (2) estimate UC probability using images alone or images plus clinical information. The IBD specialists performed task (1) under the same conditions. Their diagnostic performance was compared.

**Results:**

The median age of the patients was 36.5 and 28 years in the UC and CD groups, respectively. The diagnostic accuracy without clinical information was 75.6% for ChatGPT and 84.9% for specialists, which increased to 87.4% and 88.7% with clinical information, respectively. The odds ratios for correct diagnosis markedly increased when clinical data were used. Receiver operator curve analysis of ChatGPT showed area under the curves of 0.750 without clinical information and 0.948 with clinical information.

**Conclusion:**

ChatGPT accurately discriminated between UC and CD, with diagnostic accuracy markedly increased via the integration of clinical information, suggesting applicability in clinical practice despite being less accurate than IBD specialists.

## 1 Introduction

Ulcerative colitis (UC) and Crohn's disease (CD) are major inflammatory bowel diseases (IBD). The Global Burden of Disease Study 2019 estimated that approximately 4.9 million individuals were diagnosed with IBD in 2019 [1, 2]. UC is characterized by continuous mucosal inflammation extending proximally from the rectum to the colon, typically presenting as bloody stools. In contrast, CD can involve any segment of the gastrointestinal tract from the oral cavity to the anus, exhibiting discontinuous (“skip”) lesions with transmural inflammation, longitudinal ulcers, cobblestoning, and fistulas or strictures. Because UC and CD differ in clinical presentation, genetic background, immunopathogenesis, complications, and long‐term prognosis, early differential diagnosis is essential [3, 4, 5, 6]. Colonoscopy (CS) remains the gold standard for differentiating UC from CD [7, 8]. However, in real‐world clinical practice, this distinction can still be challenging, particularly when endoscopic findings overlap or are atypical. Although novel serologic markers, such as anti‐integrin αvβ6 autoantibodies, have recently been reported [9], widely accessible biomarkers remain limited.

Endoscopic differentiation relies on the assessment of lesion distribution, characteristic mucosal appearance, and histological features. Typical endoscopic findings of UC include diffuse and continuous inflammation extending from the rectum, with edematous mucosa, erythema, loss of vascular patterns, granularity, mucosal friability, and bleeding upon contact. Chronic disease may be accompanied by pseudopolyps or luminal strictures [5, 7, 10]. In contrast, CD typically shows patchy, discontinuous inflammation, aphthous and longitudinal ulcers, cobblestone mucosa, strictures, rectal sparing with skip lesions, and perianal disease [8, 11, 12]. Although CS is indispensable for differential diagnosis and therapeutic decision‐making, interpretation requires substantial expertise, and accurately distinguishing between UC and CD can be challenging for non‐specialists [13, 14, 15].

In gastrointestinal endoscopy, applying deep learning has rapidly expanded, with high‐accuracy convolutional neural network (CNN)‐based systems reported for polyp detection [16], early gastric cancer detection [17], and endoscopic activity assessment in UC [18]. Conversely, large language models (LLMs), such as ChatGPT, excel at integrating textual information and performing complex reasoning, but lack robust evidence to support their use as diagnostic aids in clinical practice. Recently, Levartovsky et al. conducted a pilot study in which ChatGPT‐4 evaluated real‐world endoscopic images of UC using the Mayo Endoscopic Subscore (MES), demonstrating accuracy and reproducibility comparable to those of IBD specialists [19].

This study aimed to evaluate the ability of ChatGPT to differentiate UC from CD using CS images and minimal clinical information and to compare its diagnostic performance with that of expert IBD endoscopists.

## 2 Methods

### 2.1 Study Design and Participants

This single‐center, retrospective study included patients diagnosed with UC or CD in accordance with Diagnostic Criteria and Treatment Guidelines for Ulcerative Colitis and Crohn's Disease [20] issued by the Japanese Ministry of Health, Labour and Welfare, who underwent CS for clinically active IBD at our institution between April 1, 2001, and May 31, 2025. Among patients with CD, only those with colonic disease (L2) or ileocolonic disease (L3) were included in the analysis. Clinical flares were defined based on the information from the treating physician in the medical records. The initial cohort comprised 386 patients with UC and 161 with CD. To avoid within‐patient correlations, only the first CS performed during a flare within the study period was included for each patient.

### 2.2 Selection of Colonoscopic Images and Clinical Variables

For each CS report, a general internal medicine resident preselected one representative still image depicting the most severe mucosal abnormality in the colon in accordance with a written process manual. For patients with ileocolonic disease (L3), images from the terminal ileum were excluded, and only colonic images were eligible for selection. A board‐certified physician reviewed and adjudicated eligibility. Images were excluded if they did not depict the colon or lacked active disease. Active disease in UC was defined as the presence of ≥ 1 of the following endoscopic findings: marked erythema, loss of vascular pattern, friability, erosions, spontaneous bleeding, or ulceration; these findings correspond to a Mayo endoscopic subscore (MES) of 2 or 3. Additionally, we extracted two structured binary clinical variables to provide a minimal clinical context alongside the image: continuous involvement of the rectum (yes/no) and the presence of perianal lesions (yes/no). No additional clinical, laboratory, or treatment information was provided to the readers or model.

### 2.3 Diagnostic Tasks Performed by ChatGPT and Specialist Readers

We used independent ChatGPT‐5.0 Thinking accounts with the setting “Improve the model for everyone” turned off, and a new chat was used for each study. Turning the setting “Improve the model for everyone” off means that the input given to ChatGPT‐5.0 Thinking is not used for model training and, therefore, prevents interference of the other data extraction session from the same project. Two diagnostic tasks were designed to evaluate the diagnostic performance of ChatGPT. Task 1 (binary classification) required classifying each case as UC or CD and was performed by ChatGPT and four board‐certified gastroenterologists, whereas task 2 (probability estimation) was performed only by ChatGPT and required estimating the probability that each case represented UC. For task 1, we queried ChatGPT (GPT‐5 Thinking, August 2025 version) via the enterprise API and asked four board‐certified gastroenterologists (median experience; 11.5 years, range; 7–20 years) to perform the same classification task independently. ChatGPT was instructed to classify each case as UC or CD and provide a single, non‐revised judgment, yielding a categorical label (UC or CD). Each case was evaluated using imaging only or imaging plus minimal clinical information (continuous involvement of the rectum and presence of perianal lesions). For specialist readers, to ensure balanced workload and minimize systematic bias, the 238 cases were randomly allocated among the four specialists: Specialist A evaluated 61 cases, Specialist B evaluated 62 cases, Specialist C evaluated 51 cases, and Specialist D evaluated 64 cases. Each specialist evaluated their assigned cases under both information conditions (image only and image plus clinical information), with case order randomized for each condition. The aggregate performance of the four specialists was used as the benchmark for comparison with ChatGPT. All evaluators were blinded to the reference standard diagnosis, study period, and any additional clinical information beyond the two prespecified variables (rectal continuity and perianal lesions). Consequently, four diagnostic settings were analyzed; ChatGPT‐a (image only), ChatGPT‐b (image plus clinical information), Specialist‐a (image only), and Specialist‐b (image plus clinical information). For task 2, the model was additionally asked to estimate the probability (0%–100%) that the case represented UC. Each case was evaluated once for each information condition.

### 2.4 Outcome Measures (Endpoints)

The primary endpoint of task 1 was the overall diagnostic accuracy for each evaluator (ChatGPT or specialists) under each information condition (image only vs. image plus clinical information). Secondary endpoints were class‐specific sensitivity (SN) and specificity (SP) for UC and CD, each with 95% confidence intervals (CIs), and prespecified threshold‐level positive predictive value (PPV), negative predictive value (NPV), and accuracy (ACC). The primary endpoint of task 2 was the area under the receiver operating characteristic (ROC) curve (AUC) for diagnosing UC based on model‐reported probabilities, whereas secondary endpoints were SN, SP, PPV, NPV, and ACC at the Youden‐index–derived threshold (95% CIs).

### 2.5 Reclassification Analysis After Adding Clinical Information

To quantify changes in classification induced by adding clinical information (image only vs. image plus clinical information), we prespecified UC as the positive class and calculated Δsensitivity = *P*(reclassified toward UC | true UC) − P(reclassified toward CD | true UC); Δspecificity = *P*(reclassified toward CD | true CD) − P(reclassified toward UC | true CD); net reclassification improvement (NRI) = Δsensitivity + Δspecificity; and Δbalanced accuracy = (Δsensitivity + Δspecificity)/2. Intuitively, Δsensitivity represents the proportion of true‐UC cases whose classification changes from CD to UC minus those that change from UC to CD. Δspecificity is defined analogously for true‐CD cases. For each evaluator (ChatGPT or specialist), the reclassification direction was defined at the case level by comparing paired labels between image only and image plus clinical information conditions. Stratified bootstrap (stratified by true diagnosis: UC vs. CD) with 2000 bootstrap resamples (B = 2000) was used to estimate 95% CIs for Δsensitivity, Δspecificity, NRI, and Δbalanced accuracy, preserving paired before/after labels within each evaluator. All summary measures were recalculated for each bootstrap sample. CIs were defined as the 2.5th and 97.5th percentiles of the resulting bootstrap distributions.

### 2.6 Statistical Analysis

Analyses were performed using R version 4.5.1 (R Foundation for Statistical Computing, Vienna, Austria). For task 1, the performance of each evaluator and condition is summarized using 2 × 2 tables. PPV, NPV, and ACC were compared between evaluators and across paired conditions using Chi‐square tests and logistic regression models with robust standard errors, including fixed effects for evaluator (ChatGPT vs. specialists), information condition (image only vs. image plus clinical information), and their interaction, and the results were reported as odds ratios (ORs) with 95% CIs. For task 2, ROC curves were constructed from UC probabilities, AUCs with 95% CIs were estimated using DeLong's method, and paired‐condition comparisons were performed using DeLong's test. The Youden index cutoff was defined as the operating threshold at which the PPV, NPV, and ACC were calculated. The exact binomial 95% CIs were reported for these measures. Statistical significance was set at *p* < 0.05.

### 2.7 Ethics

This study was approved by the Institutional Review Board of Kobe University Hospital (approval no. B250123). Given the retrospective design and use of de‐identified images, the requirement for written informed consent was waived. Instead, consent was obtained via an opt‐out procedure.

## 3 Results

### 3.1 Patient Characteristics

**FIGURE 1:**
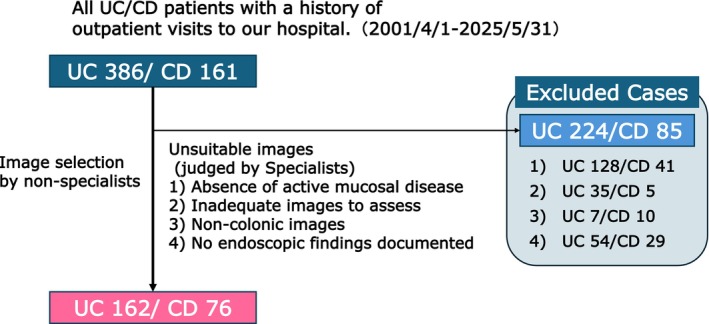
STARD flow diagram of patient screening, eligibility, and inclusion (final analysis: UC = 162; CD = 76).

One image per case was extracted from the CS reports of 386 patients with UC and 161 with CD. Images were required to show active disease corresponding to MES2 or three in patients with UC. After excluding cases that did not meet the eligibility criteria, the remaining cases were included in the final analysis. The final dataset study included 238 patients (UC, *n* = 162, median age; 36.5 years, CD, *n* = 76 median age; 28 years) (Table 1; Figure 1). The male‐to‐female ratio was 87/75 in UC and 50/26 in CD. Among patients with UC, MES 2 and 3 accounted for 74.1% (*n* = 120) and 25.9% (*n* = 42), respectively. The median SES‐CD was 12.5.

**TABLE 1:** Baseline characteristics of UC and CD patients.

UC	*n* = 162
Age, years, median (range)	36.5 (6–79)
Gender Male/female (%)	87 (53.7%)/ 75 (46.3%)
MES median(range)	2 (2–3)
MES (%)	
MES 2	120 (74.1%)
MES 3	42 (25.9%)
Anal lesions	0 (0%)
Rectal skip	10 (6.2%)
**CD**	n **= 76**
Age, years, median (range)	28 (8–73)
Gender Male/Female [% (n)]	50 (65.8%) / 26 (34.2%)
SES‐CD median (range)	12.5 (4–30)
SES‐CD (%)	
Mild [3–6]	15 (19.7%)
Moderate [7–15]	33 (43.4%)
Severe [16‐]	28 (36.8%)
Anal lesions	26 (34.2%)
Rectal skip	69 (90.8%)

### 3.2 ChatGPT‐Based Classification of UC and CD

**FIGURE 2:**
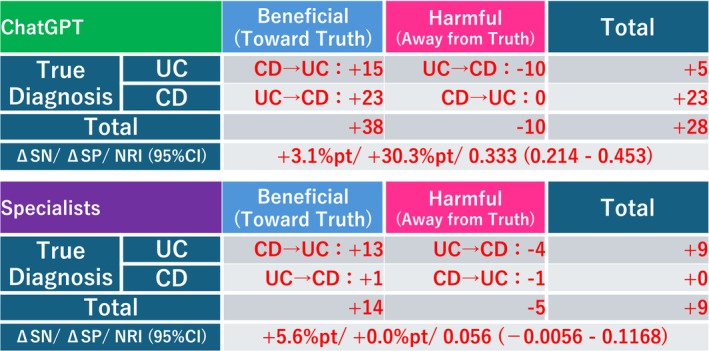
Diagnostic performance and the odds ratios of ChatGPT and specialists. ACC, accuracy; NPV, negative predictive value; OR: the odds ratio; PPV: positive predictive value; SN, sensivity; SP, specificity.

Without clinical information, ChatGPT correctly classified 139 of the 162 UC images (85.8%) and 41 of the 76 CD images (53.9%). With the clinical information, the performance improved to 144 (88.9%) UC images and 64 (84.2%) CD images, and the overall accuracy increased from 75.6% (180/238) to 87.4% (208/238) (Supporting Information, Figure 2). Sensitivity, specificity, and overall accuracy were calculated from four 2 × 2 contingency tables, one for each combination of evaluator (ChatGPT or specialist) and clinical context (images only vs. images plus clinical information) (Figure 2). Under the image‐only condition, the accuracy of discriminating UC from CD was 75.6% for ChatGPT and 84.9% for specialists; if clinical information was added, the accuracy increased to 87.4% and 88.7%, respectively. If clinical information was available, the ORs for correct vs. incorrect classifications, derived from the same tables, increased for both evaluators. For ChatGPT, the ORs were 7.08 with images only and 42.70 with images plus clinical information; for specialists, the corresponding ORs were 26.6 and 51.5, respectively (all *p* < 0.001).

### 3.3 Impact of Adding Minimal Clinical Information on Image‐Based Classification

**FIGURE 3:**
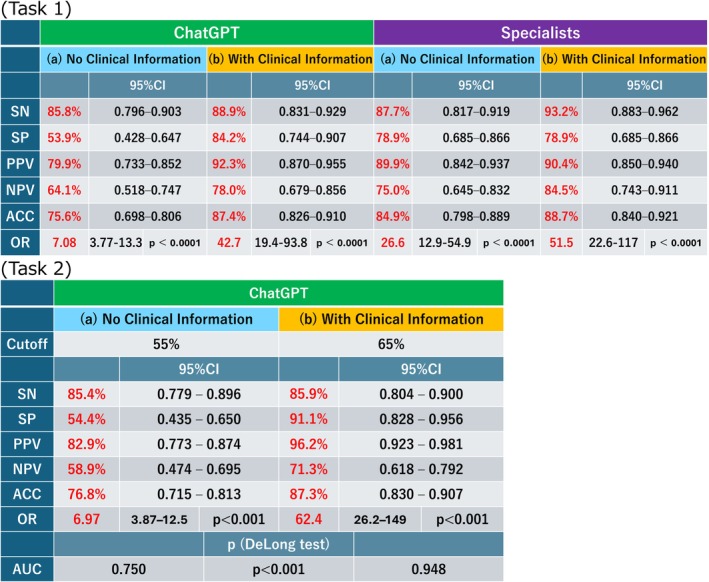
For ChatGPT and Specialists: (1) counts of CD → UC (benefit) and UC → CD (harm) in true‐UC cases, and (2) counts of UC → CD (benefit) and CD → UC (harm) in true‐CD cases. The total row shows overall beneficial vs. harmful counts and the net. Definition: Beneficial reclassification = toward the true diagnosis; Harmful reclassification = away from the true diagnosis. Clinical fields: Presence of perianal lesions and discontinuous involvement from the rectum.

We examined the impact of adding minimal clinical information to the ChatGPT image‐based classification and compared the resulting reclassification patterns with those of specialist readers. For ChatGPT, 15 CD‐to‐UC corrections and 10 UC‐to‐CD incorrect reclassifications occurred among the true‐UC cases (*n* = 162), yielding a net improvement in five cases. Among the true‐CD cases (*n* = 76), 23 UC‐to‐CD reclassifications and no CD‐to‐UC flips occurred, corresponding to a net improvement in 23 cases. Overall, the beneficial vs. harmful reclassification rates were 38 vs. 10, respectively, corresponding to a net improvement of 28 cases (Figure 3). For specialists, 13 CD‐to‐UC and four UC‐to‐CD reclassifications occurred among true‐UC cases (net improvement of nine cases), and one UC‐to‐CD and one CD‐to‐UC reclassification occurred among true‐CD cases (no net change). The beneficial vs. harmful reclassifications were 14 vs. five, respectively, corresponding to a net improvement of nine cases. Corresponding changes, taking UC as the positive class, were Δsensitivity +3.1% points, Δspecificity +30.3% points, and NRI 0.333 (95% CI; 0.214–0.453) for ChatGPT and Δsensitivity +5.6% points, Δspecificity 0.0% points, and NRI 0.056 (95% CI; −0.0056–0.1168) for specialists; all 95% CIs were estimated via stratified bootstrap. Overall, these reclassification metrics indicate that adding minimal clinical information meaningfully improved the ChatGPT classification of UC vs. CD, whereas its impact on specialist readers was modest. Notably, incorporating even limited clinical context may enable ChatGPT to achieve diagnostic performance comparable to specialist assessment.

### 3.4 Image Features of Patients With UC Misclassified as CD

**FIGURE 4:**
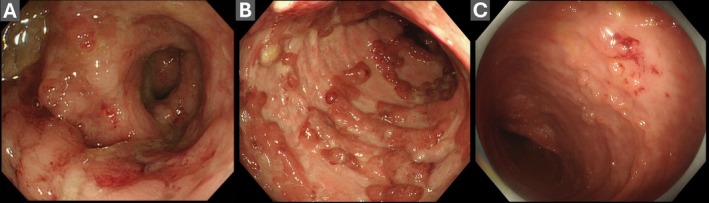
Representative ulcerative colitis (UC) cases misclassified as Crohn's disease (CD). (A) Shallow linear erosions; (B) cobblestone‐like mucosa associated with pseudopolyposis; (C) apparently patchy distribution. Across all panels, a continuous background of diffuse inflammation is present, with no deep undermined ulcers, definite skip lesions, fistulas, or strictures.

Among the 18 UC cases misclassified as CD under the image and clinical information conditions, all images showed a continuous background of UC‐typical inflammation—diffuse erythema, edema, granular mucosa, loss of vascular pattern, and bleeding upon contact, in which Crohn‐like features were superimposed: (1) shallow longitudinal/serpiginous erosions formed by confluent superficial lesions, (2) a cobblestone‐like appearance due to pseudopolyposis, and (3) segments with apparent patchiness due to mixed activity and healing. Classic hallmarks strongly suggestive of CD, discrete deep undermined ulcers, true skip lesions across the normal mucosa, strictures, fistulous openings, and perianal disease, were uniformly absent in these cases (Figure 4).

### 3.5 Probabilistic Analysis of UC Diagnosis by ChatGPT Using Endoscopic Images of UC and CD

**FIGURE 5:**
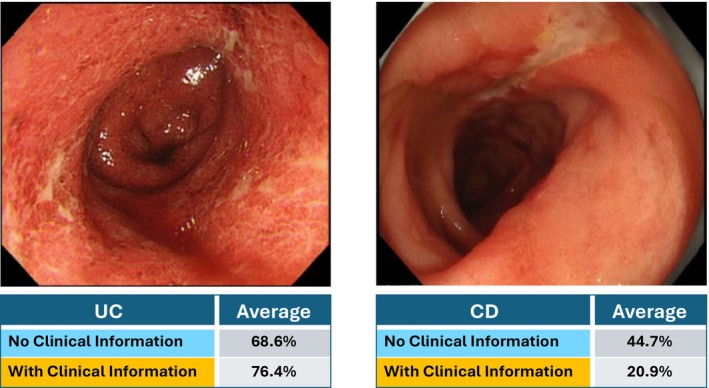
Mean probabilities estimated by ChatGPT for ulcerative colitis (UC) with and without clinical information, using images of UC and Crohn's disease (CD).

**FIGURE 6:**
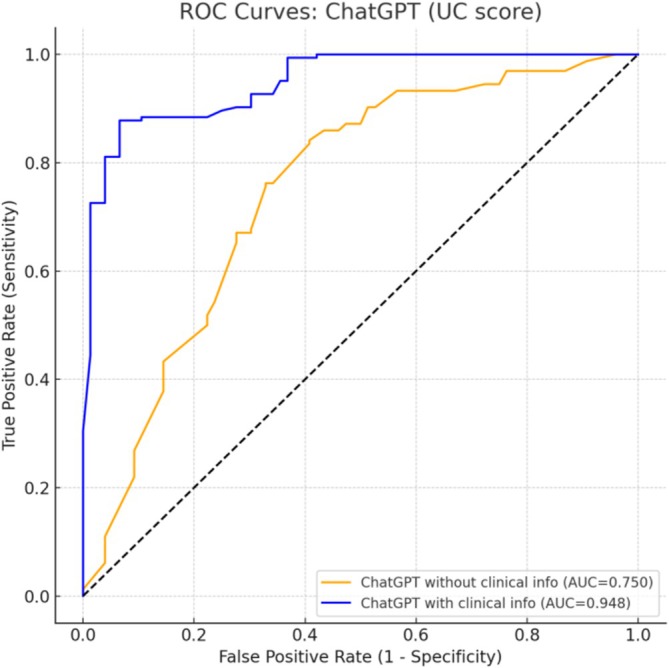
ROC curves for ChatGPT demonstrating the diagnostic performance of ChatGPT in predicting UC using endoscopic images alone versus images with additional clinical information.

In task 2, ChatGPT estimated the probability that each endoscopic image represented UC. The mean estimated probability for the UC images was 68.6% and 76.4% without and with clinical information, respectively. If the CD images were input, the mean estimated probabilities for UC were 44.7% and 20.9%, respectively (Figure 5). Because task 2 required estimating the probability that an image represented UC, the CD images naturally received lower probability estimates. The diagnostic performance in predicting UC was assessed using ROC analysis. The AUC was 0.750 if only endoscopic images were provided and increased to 0.948 if clinical information was included (Figure 6). At the optimal cutoff values determined using the Youden index (55% without and 65% with clinical information), the overall accuracies were 76.8% and 87.3%, respectively. The improvement in the AUC was statistically significant according to DeLong's test (*p* < 0.001).

## 4 Discussion

Endoscopic differentiation between UC and colonic‐type CD has traditionally relied on the expert judgment of gastroenterologists. Recently, CNN‐based image‐specific AI models have been developed to classify IBD subtype [21, 22]. However, many of these models were trained using images derived from a single institution, raising concerns about their generalizability and external validity across institutions.

This study evaluated the ability of ChatGPT to differentiate UC from CD using a single endoscopic image supplemented with minimal clinical information and achieved an accuracy of 87.4% if lesion distribution and continuity were provided. Although this accuracy is slightly lower than that of task‐specific deep‐learning models, including a report showing 99.1% per‐patient accuracy [22] or ResNeXt‐101‐based CNN outperforming multiple endoscopists [21] and slightly below the classical diagnostic accuracy of colonoscopic assessment for IBD overall (approximately 89%) [23], the performance is comparable to that of experienced endoscopists and surpasses that of novices. The novelty and clinical significance of this study lie in demonstrating that a general‐purpose LLM can flexibly integrate a single image with minimal clinical information to achieve near‐expert‐level UC/CD discrimination.

Notably, the diagnostic accuracy of ChatGPT significantly improved if clinical information was added, from 53.9% to 84.2% for CD, with an NRI of 0.333. In contrast, although clinical information slightly increased the expert endoscopists' accuracy from 84.9% to 88.7%, the NRI was only 0.056, indicating a much smaller incremental benefit. This suggests that expert clinicians have already achieved high diagnostic discrimination based on images alone, whereas ChatGPT derives substantial additional benefits from the clinical context. Consistently, a multimodal model integrating endoscopic images and clinical data outperformed an image‐only CNN in diagnosing cytomegalovirus colitis complicating UC [24], and studies in the gastrointestinal field have emphasized the importance of integrating clinical information for AI‐based diagnosis [25]. These observations support the view that LLMs, such as ChatGPT, function as image pattern‐recognition systems and “multimodal integrators” capable of probabilistic reasoning across textual and visual modalities.

Detailed evaluation of misclassified cases is essential for understanding the inherent limitations of endoscopic diagnosis and the characteristics of “difficult cases.” In this study, 18 UC cases were misclassified as CD by ChatGPT even after clinical information was added. These cases showed typical features of continuous UC inflammation, such as diffuse erythema, edema, granularity, loss of vascular pattern, and bleeding upon contact, but exhibited overlapping “Crohn‐like” features, including shallow longitudinal erosions and a cobblestone‐like appearance due to clusters of pseudopolyps. Importantly, no definitive CD features, such as deep ulcers and clear skip lesions separated by normal mucosa, were present. In clinical practice, such cases are frequently encountered at the UC‐colonic CD boundary and often labeled “IBD unclassified (IBD‐U)” or “indeterminate colitis.” [26, 27, 28] A cohort study reported that approximately 80% of IBD‐U cases were ultimately reclassified as UC or CD during long‐term follow‐up [29]. These findings underscore that single‐time‐point endoscopy has inherent limitations in disease classification, posing a challenge for AI and human specialists.

This study had several limitations. First, this single‐center retrospective analysis was susceptible to bias due to local endoscopy practices and equipment at Kobe University, and external validation in multi‐center settings with more diverse imaging quality is warranted before broader clinical application. Second, representing each case with a single image inherently precludes assessment of lesion distribution across the entire colon. Third, this study evaluated subtype classification between UC and CD in patients with confirmed IBD, rather than diagnosing IBD de novo. The performance of ChatGPT in distinguishing IBD from non‐IBD mimics, such as infectious, ischemic, or tuberculous colitis, remains to be evaluated in future studies. Finally, the two clinical variables used in this study inherently have pathognomonic features that strongly predict UC or CD. Given their strong discriminative power, it cannot be ruled out that these variables may themselves function as determinants of diagnosis, rather than serving merely as complementary information to image‐based analysis. However, this study had several strengths. A head‐to‐head comparison with IBD specialists contextualizes LLM performance. Diagnostic gains were quantified using robust statistical metrics, and a detailed analysis of misclassified cases highlighted the inherently challenging domains of the LLM‐based diagnosis.

Overall, this study demonstrated that ChatGPT can differentiate UC from CD using only a single colonoscopic image and minimal clinical information, achieving reasonable performance from images alone and improving to a near‐expert level if clinical context is added, thereby supporting IBD diagnosis and complementing clinical decision‐making in appropriate settings.

## Funding

The authors have nothing to report.

## Conflicts of Interest

The authors declare no conflicts of interest.

## Supporting information


**Data S1:** Contingency tables (2 × 2) of diagnostic classifications by ChatGPT and specialists with and without clinical information.

## Data Availability

The data that support the findings of this study are available on request from the corresponding author. The data are not publicly available due to privacy or ethical restrictions.
