# Supplementary material for: ChatGPT‐Assisted Image Interpretation for Inflammatory Bowel Diseases: Ulcerative Colitis and Crohn's Disease
Source: JGH Open. 2026 Apr 2;10(4):e70396. doi: 10.1002/jgh3.70396 (PMC13045225; doi:10.1002/jgh3.70396)
Supplement: Supplementary file 1 — Data S1: Contingency tables (2 × 2) of diagnostic classifications by ChatGPT and specialists with and without clinical information. [file JGH3-10-e70396-s001.pptx]

## Slide 1
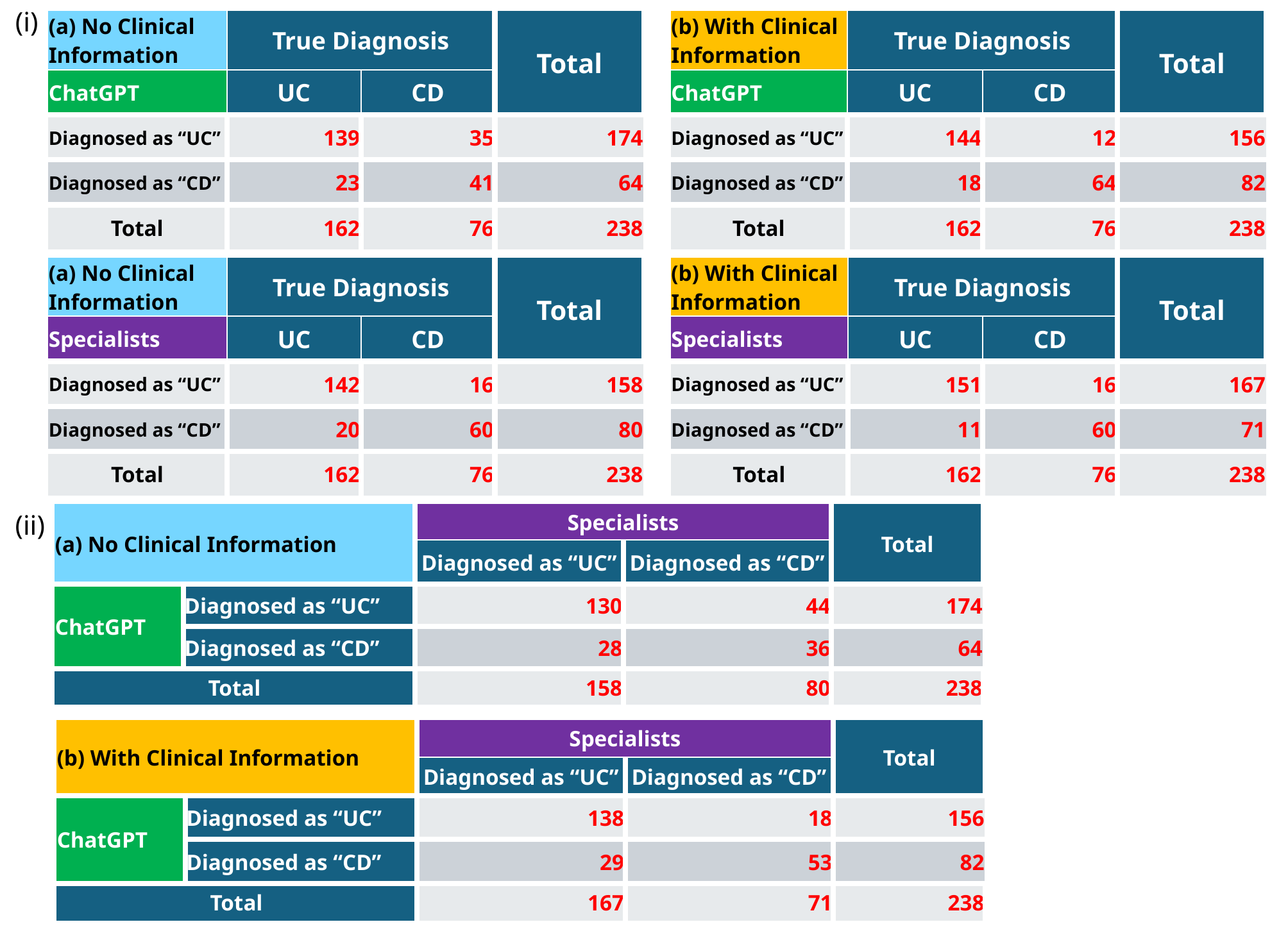

(i)
| (a) No Clinical Information | True Diagnosis | | Total |
| --- | --- | --- | --- |
| ChatGPT | UC | CD | |
| Diagnosed as “UC” | 139 | 35 | 174 |
| Diagnosed as “CD” | 23 | 41 | 64 |
| Total | 162 | 76 | 238 |
| (b) With Clinical Information | True Diagnosis | | Total |
| --- | --- | --- | --- |
| ChatGPT | UC | CD | |
| Diagnosed as “UC” | 144 | 12 | 156 |
| Diagnosed as “CD” | 18 | 64 | 82 |
| Total | 162 | 76 | 238 |
| (a) No Clinical Information | True Diagnosis | | Total |
| --- | --- | --- | --- |
| Specialists | UC | CD | |
| Diagnosed as “UC” | 142 | 16 | 158 |
| Diagnosed as “CD” | 20 | 60 | 80 |
| Total | 162 | 76 | 238 |
| (b) With Clinical Information | True Diagnosis | | Total |
| --- | --- | --- | --- |
| Specialists | UC | CD | |
| Diagnosed as “UC” | 151 | 16 | 167 |
| Diagnosed as “CD” | 11 | 60 | 71 |
| Total | 162 | 76 | 238 |
(ii)
| (a) No Clinical Information | | Specialists | | Total |
| --- | --- | --- | --- | --- |
| | | Diagnosed as “UC” | Diagnosed as “CD” | |
| ChatGPT | Diagnosed as “UC” | 130 | 44 | 174 |
| | Diagnosed as “CD” | 28 | 36 | 64 |
| Total | | 158 | 80 | 238 |
| (b) With Clinical Information | | Specialists | | Total |
| --- | --- | --- | --- | --- |
| | | Diagnosed as “UC” | Diagnosed as “CD” | |
| ChatGPT | Diagnosed as “UC” | 138 | 18 | 156 |
| | Diagnosed as “CD” | 29 | 53 | 82 |
| Total | | 167 | 71 | 238 |
